# Does Nocturnality Drive Binocular Vision? Octodontine Rodents as a Case Study

**DOI:** 10.1371/journal.pone.0084199

**Published:** 2013-12-31

**Authors:** Tomas Vega-Zuniga, Felipe S. Medina, Felipe Fredes, Claudio Zuniga, Daniel Severín, Adrián G. Palacios, Harvey J. Karten, Jorge Mpodozis

**Affiliations:** 1 Departamento de Biología, Facultad de Ciencias, Universidad de Chile, Santiago, Chile; 2 Centro Interdisciplinario de Neurociencia de Valparaíso, Facultad de Ciencias, Universidad de Valparaíso, Valparaíso, Chile; 3 Department of Neurosciences, School of Medicine, University of California San Diego, La Jolla, California, United States of America; University of Lethbridge, Canada

## Abstract

Binocular vision is a visual property that allows fine discrimination of in-depth distance (stereopsis), as well as enhanced light and contrast sensitivity. In mammals enhanced binocular vision is structurally associated with a large degree of frontal binocular overlap, the presence of a corresponding retinal specialization containing a fovea or an area centralis, and well-developed ipsilateral retinal projections to the lateral thalamus (GLd). We compared these visual traits in two visually active species of the genus Octodon that exhibit contrasting visual habits: the diurnal *Octodon degus*, and the nocturnal *Octodon lunatus*. The *O. lunatus* visual field has a prominent 100° frontal binocular overlap, much larger than the 50° of overlap found in *O. degus*. Cells in the retinal ganglion cell layer were 40% fewer in *O. lunatus* (180,000) than in *O. degus* (300,000). *O. lunatus* has a poorly developed visual streak, but a well developed area centralis, located centrally near the optic disk (peak density of 4,352 cells/mm^2^). *O. degus* has a highly developed visual streak, and an area centralis located more temporally (peak density of 6,384 cells/mm^2^). The volumes of the contralateral GLd and superior colliculus (SC) are 15% larger in *O. degus* compared to *O. lunatus*. However, the ipsilateral projections to GLd and SC are 500% larger in *O. lunatus* than in *O. degus*. Other retinorecipient structures related to ocular movements and circadian activity showed no statistical differences between species. Our findings strongly suggest that nocturnal visual behavior leads to an enhancement of the structures associated with binocular vision, at least in the case of these rodents. Expansion of the binocular visual field in nocturnal species may have a beneficial effect in light and contrast sensitivity, but not necessarily in stereopsis. We discuss whether these conclusions can be extended to other mammalian and non-mammalian amniotes.

## Introduction

Among vertebrates, the size, shape and position of the eyes and their orbits exhibit a high degree of variability, ranging from the small, unilaterally placed eyes of the flatfishes (*Achirus lineatus*), to the large, highly convergent frontal eyes of humans and other apes [1], [2]. Peculiar (from a human perspective) placement of the eyes is not exceptional among fishes, perhaps reflecting the high diversity of morphological features present in these taxa. For instance, the eccentric lateral placement of the eyes of the hammerhead sharks (Sphyrnidae) endows them with a 360° span of the visual field in the horizontal and vertical dimensions, while retaining at the same time a significant amount of binocular overlap [3].

Among amniotes it is a well-established fact that convergent, more frontally oriented eyes, grant higher degrees of binocular overlap, while lateralized placement of eyes result in narrow binocular visual fields [1], [4], [5]. The degree of ocular convergence, in turn, is associated with differences in other functional aspects of the visual system, such as the presence and position of retinal specializations and the relative emphasis of the different retinal projections. In mammals, the position of the area centralis (the retinal specialization that subserves the binocular area of the visual field) varies from temporal in species with lateralized eyes to pericentral in species with frontal eyes [6]–[8]. Furthermore, in birds and mammals, the relative size of the visual thalamofugal projection is larger in species with frontally-oriented eyes [4], [9], [10]. In mammals the retinal ipsilateral projections to the thalamic nucleus geniculatus lateralis pars dorsalis (GLd), the visual pathway that mediates functional binocular vision, is positively correlated with the degree of binocular overlap [11]–[14].

Various observations show that among amniotes feeding behavior influences eye orientation. Aerial and terrestrial predators (e.g. Felidae, Strigiformes and some Caprimulgiformes) have a larger degree of binocular overlap than their prey (ranging from large herbivores to small ground feeding birds) [4], [15]–[17]. In addition, comparative studies indicate that irrespective of their feeding habits, nocturnal animals also exhibit a high degree of binocularity. Different taxa of nocturnal ammniotes such as the grey-headed flying fox (Megachiroptera), the Galago (Galagidae), and even the nocturnal parrot Kakapo (Strigopidae), can be cited as representative examples of this tendency [18]–[20].

Given these observations, enhanced binocular vision has been associated with several ecological/behavioral factors, such as nocturnality, predatory behavior and arboreal substrate. Although several comparative studies on the topic have been published [8], [15], [21]–[23], direct test of these ecological factors on the structural traits underlying binocular vision is lacking, likely due to the scarcity of closely related species that differ in visual abilities and life histories.

Hystricognath rodents belonging to the endemic South American Octodontidae family can be considered a natural experiment, as it includes species with markedly different visual habits. The genus Octodon, endemic to central Chile, is of particular interest because it contains two closely related surface dwelling species with opposite visual habits: the diurnal *O. degus* and the nocturnal *O. lunatus*. Phylogenetic analyses suggest that these species split from a degus-like ancestor two-three million years ago (Pliocene) [24], [25].

The goal of this study is to compare the main structural features associated with binocular vision in these two closely related species. In particular, we studied the shape and position of the eyes and their orbits, the extent and position of the visual field, the distribution and densities of the retinal ganglion cells, and the characteristics of the retinal central projections. The results indicate that the adoption of a nocturnal visual habit indeed leads to an enhancement of binocular vision in the nocturnal *O. lunatus.* We discuss whether this conclusion can be extended to other mammalian and non-mammalian amniotes.

## Methods

Three *Octodon degus* and three *Octodon lunatus* weighing 250–300 gr. and including both males and females were captured in the wild and kept in a standard animal cage.

All animals were treated following the Guide for the Care and Use of Laboratory Animals of the National Institutes of Health. The experimental procedures were approved by the Faculty of Sciences of University of Chile Ethics Committee (Permit 29-9-011). All efforts were made to minimize animal suffering.

In addition, skulls from two adult individuals of each species, and a sample of isolated fixed eyes of both species were used in this study. These materials were available from the collection of Dr. Raul Sobrero, at Universidad Católica de Chile.

### Eye Size Measurements

Eye measurements were carried out in a sample of seven eyes taken from four specimens of *O. lunatus* and six eyes taken from three specimens of *O. degus*. The axial length (AL), transverse length (TL) and corneal diameter (CD) of these eyes were measured using a digital caliper under a dissection microscope.

### Determination of Orbit Convergence Angle

Following Hessy (2004), the orbit convergence angle was defined as the dihedral angle formed by the intersection of the ocular orbit plane and the sagittal plane. To determine this angle, the skulls of two individuals from each species were mounted in a head holder at the rat stereotaxic position [26]. The x, y and z coordinates of the various landmarks required to determine these planes (for a detailed description see Hessy, 2004) were measured using a fine probe mounted in a 3-axis stereotaxic manipulator (50 µm precision). Once the planes were determined, the convergence angle was calculated using a simple, custom-made algorithm implemented in commercial software (Igor Pro 6).

### Visual Field Measurements

**Figure 1:**
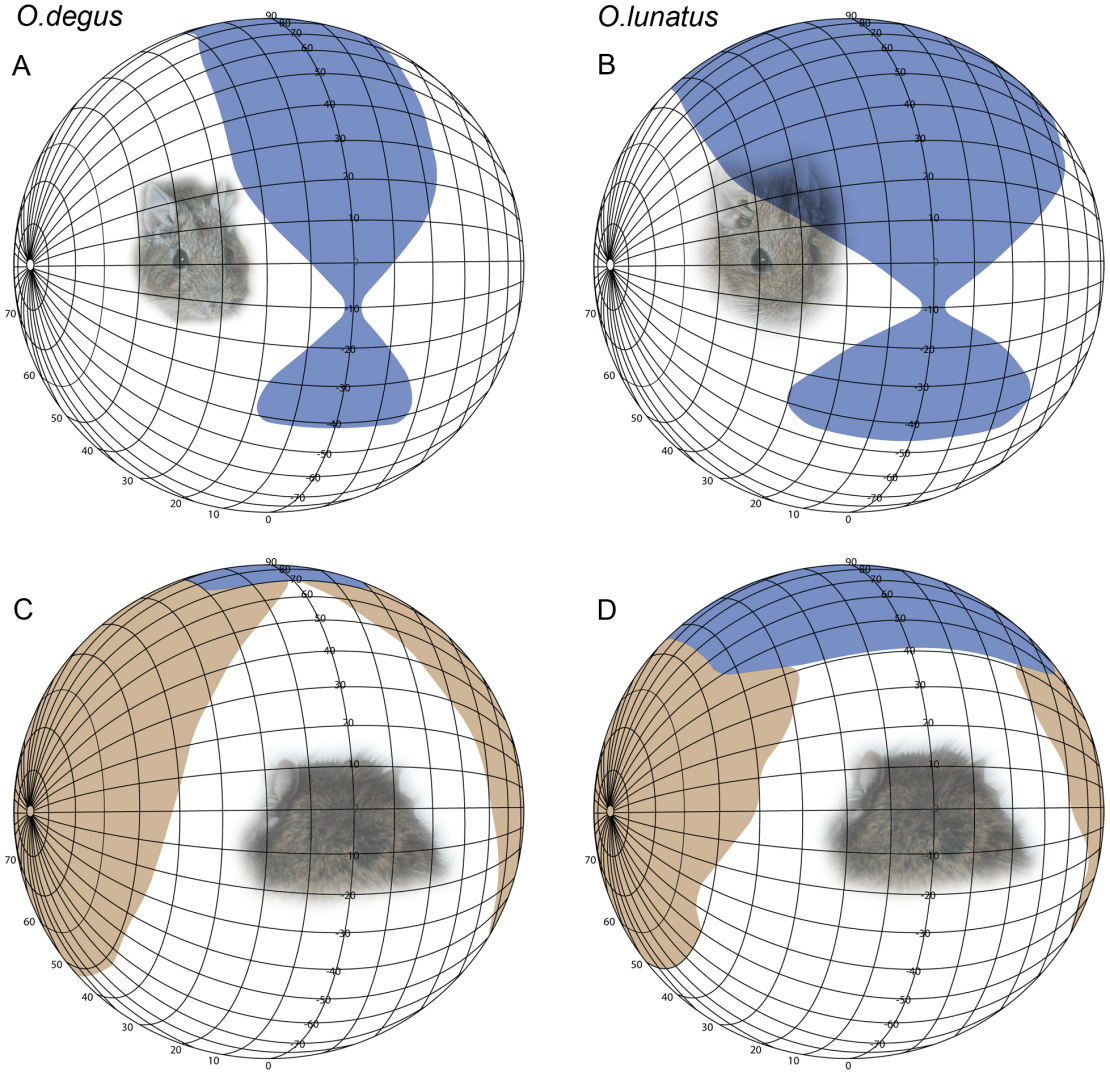
Binocular and monocular fields. Perspective view of the binocular visual field (blue) in the diurnal *O. degus* and nocturnal *O. lunatus*. Maximum binocular overlap in *O. degus* is 50° (A). The nocturnal rodent *O. lunatus* has a maximum binocular overlap of 100° (B). Perspective view of the posterior monocular visual field (brown) (C,D). In *O. degus* the monocular field is 170°. The nocturnal rodent *O. lunatus* has a monocular visual field of 190°. The diagram uses the conventional latitude and longitude system.

Measurements of visual fields were made in animals anesthetized with a mixture of ketamine (120 mg/kg IP) and xylazine (4 mg/kg IP), mounted in a stereotaxic head holder inside a campimeter. The head was held at the center of the visual perimeter so that the parpebral fissures coincided with the 0° of the rotation axis of the campimeter. This orientation is similar to the natural resting head position of these animals. The perimeter’s coordinates followed a conventional latitude and longitude system. This coordinate system was used for the presentation of visual field data (Figure 1). The eyes were examined using an ophthalmoscope reflex technique. For each eye the visual fields were determined measuring the limits of the nasal and temporal reflected retinas. To avoid ocular movements during the measures, eyes were paralyzed locally with intraorbital injections of Lidocaine.

### Preparation of Retinal whole Mounts and Ganglion Cell Layer Counts

Following fixation (transcardial perfusion, see below)**,** eyes were enucleated and washed in PBS 0.1M. Retinae were carefully dissected from their underlying pigmented layer and the optic nerves were severed just beneath their retinal attachment. The isolated retinas were washed again in PBS and mounted on moist gelatinized slides.

Once defatted, retinas were stained with 1% cresyl violet for four minutes, after which they were dehydrated through an ascending alcohol series, cleared in xylene and cover-slipped with permount.

Following Pettigrew [27], whole mounted retinas were drawn on a sheet of graph paper using an overhead projector. Care was taken to align the side of the microscope slide to the graph paper grid. The X-Y coordinates of a number of landmarks on the retinas were noted on the drawing so that all subsequent counts could be transferred to the drawing from the stage micrometer.

Under microscopic observation, cells in the ganglion cell layer were counted in a 125×125 µm optic grid. Counts were made at 1.0 mm intervals across the whole retina, except for the high density area centralis region, in which counts were made at 0.5 mm intervals. These counts were converted to ganglion cells layer per square millimeter (GCL/mm^2^) and isodensity contours were drawn on graph paper of the flat mounted retina. The total ganglion cell layer population was estimated by using the mean cell densities for each isodensity curves (except for the area centralis where the value of the contouring AC isodensity curve was used) and multiplying those values by the respective areas (in mm^2^):

(Where *A_u_* are the isodensity areas; *d_inner_*, *d_outer_* the cell densities for the isodensity contours confining each area, respectively; *d_ac_* is the isodensity contour of the area centralis; *j* is isodensity contours).

### Labeling of Retinal Projections

Six animals (three *O. degus* and three *O. lunatus*) were anesthetized as previously mentioned, and given intraocular injections (12 µL, left eye) of 0.5% Cholera toxin subunit B (CTB, List Biological Laboratories) mixed with 1% DMSO.

After a seven-day survival period, the animals were deeply anesthetized with Ketamine (120 mg/kg IP) and xylazine (4 mg/kg IP), and perfused through the heart with approximately 500 mL of saline, followed by 500 mL of 4% paraformaldehyde in PB (PFA/PB).

After removal from the skull, each brain was postfixed overnight in PFA/PB and then transferred into a solution of 30% sucrose/PB until it sank. The brains were cut in coronal, sagittal and horizontal planes at 30 µm and processed with immunohistochemical techniques to reveal CTB distribution with the avidin-biotin-peroxidase method. Briefly, sections were incubated with antibodies against CTB (made in goat, diluted 1∶20.000), followed by biotinylated secondary antibodies (Vector Laboratories); anti-goat IgG (made in rabbit, diluted 1∶200). Avidin-coupled peroxidase (Vector ABC kit) was then used with diaminobenzidine as the final chromogen. A one-in-four series of sections was examined for CTB and was later counterstained with Giemsa to reveal cell bodies. A separate one-in-four series of sections was left uncounterstained. A third series was stained for Nissl substance.

### Relative Volume of Retinal Terminals

Volume measurements were made in the CTB-Giemsa series. For this purpose we took high-resolution images with a scanning CCD camera (Leaf System, Inc., Southborough, MA) equipped with a 120 mm macro lens. Image processing was done in Adobe Photoshop CS3 Extended. Volumes were calculated using the public domain NIH image analysis software ImageJ with Volumest plugin [28]. Areas of entire selected nuclei were measured across transverse, sagittal and horizontal sections, and multiplied by section thickness (30 µm) including the sampling interval of every fourth section. We measured the contralateral/ipsilateral retinal labeling terminals volume of GLd, SC, and the contralatral retinal labeling terminals volume of the Suprachiasmatic nucleus (SCN) and of the Medial Terminal nucleus (MTN). To account for allometric effects, we divided the volume of a given structure by brain volume. To achieve this, brains were weighed to the nearest milligram. Brain volume was calculated by dividing brain mass by fixed brain tissue density (1.036 g/mm^3^) [29]. Thus, volumes of the measured structures are expressed as relative to the brain volume.

### Statistical Analysis of Retinal Terminals Volumes

Due to our small sample size, we used non-parametric statistics (i.e. Kruskal-Wallis and Mann Whitney *U-*tests) to carry out comparison between species estimates. The α-level for all tests was set to 0.05.

## Results

### Visual Field Measurements

The size and shape of the visual field are both dynamic features that vary continuously according to the dynamics of eye movements. In particular, conjugated convergent or divergent eye movements may greatly increase or decrease the extent of binocular overlap. Since these variations are technically difficult to estimate, most studies in the literature, including the present one, report measurements taken in the relaxed, akinetic state of eyes, which therefore serve as a reference for comparative purposes. We took visual field measurements on two individuals of each species. Differences between individuals of the same species were minimal (10%) when compared to the differences between species (100%). Because of this result and our limited sample size, here we present the results obtained from one representative individual from each species.

The monocular visual field of *O. degus* had a maximum extension of 170° in the naso-temporal axis, spanning from −25° frontally (contralateral hemisphere) to 145° caudally (ipsilateral hemisphere). Consequently, the region of maximum binocular overlap had an extension of 50°, and its location was dorso-rostral with respect to the visual field, between 30° and 60° of vertical elevation. The minimal binocular overlap was situated at −10° in the vertical axis, corresponding to the projection of the tip of the snout. Towards the dorso-caudal portion of the visual field the binocular overlap reached up to 45° and extended until 110° of the vertical axis. Towards the rostro-ventral portion, the binocular overlap reached 20° and extended until −42° of the vertical axis (Figure 1).

Compared with *O. degus*, *O. lunatus* has a slightly larger (190°), but clearly more frontal monocular visual field that spans from −50° frontally (contralateral hemisphere) to 140° caudally (ipsilateral hemisphere). Due to this frontal expansion, the region of maximum binocular overlap of *O. lunatus* was two times larger than that of *O. degus* (up to 100° width), and was situated between 50° and 70° of the vertical axis. The minimal binocular overlap was also located at −10° in the vertical axis. Towards the dorso-caudal visual field the binocular overlap was also larger than *O. degus*’s, reaching up to 90° and extending until 140° of the vertical axis. Towards the rostro-ventral visual field the binocular overlap reached 35° and extended until −45° of the vertical axis (Figure 1).

### Eye Size and Orbit Convergence

The eyes of *O. lunatus* were slightly larger (9% for both AL and TL) but of approximately equal shape (same LT/LA ratio) than the eyes of *O. degus* (Table 1). Interestingly, *O. lunatus* corneal diameter was 20% larger. Therefore, CD/AL ratio was also about 10% larger in *O. lunatus*.

**Table 1:** Eye measurements.

Species	TL	CD	AL	TL/AL	CD/AL	CD/TL
*O.lunatus*	8,37 (±0,26)	7,41 (±0,51)	8,17 (±0,49)	1,03 (±0,06)	0,91 (±0,04)	0,89 (±0,05)
*O.degus*	7,74 (±0,18)	6,24 (±0,26)	7,42 (±0,16)	1,04 (±0,04)	0,84 (±0,03)	0,81 (±0,04)

Calculated values for the eye measurements (n = 13) in *O. degus* and *O. lunatus* respectively.

TL =  Transverse longitude; AL =  axial longitude; CD = corneal diameter.

Convergence angles were nearly identical for individuals of the same species, but differed between species. We estimated a 41° convergence angle in *O. degus*, whereas *O. lunatus* features a more frontalized 50° angle. Both measurements agree with the values expected from the correlation between visual field overlap and orbit convergence in mammals [1].

### Distribution and Density of Cells in the Ganglion Cell Layer

**Figure 2:**
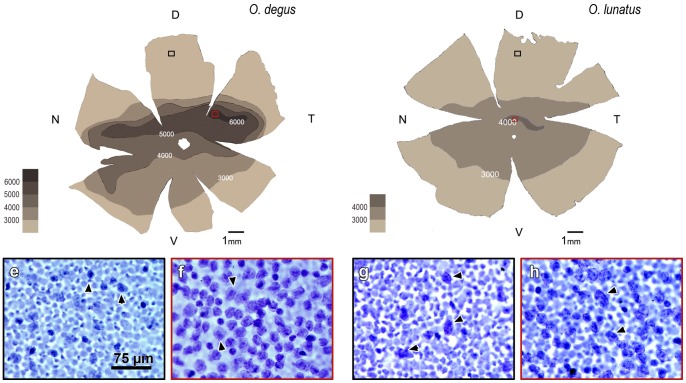
Retinal wholemounts. Ganglion cell layer (GCL) density map for the left retina of *O. degus* and *O. lunatus* (representative animal). Area centralis is located more centrally in the nocturnal *O. lunatus* compared to diurnal *O. degus*. Black and red rectangles represent insets of a dorsal and a central retinal area, respectively. Inset e: GCL density (*O. degus*) in the periphery of the dorsal retina, less than 2,000 cells/mm^2^ Inset f: GCL density (*O. degus*) in the area centralis located temporally, peak of 6,384 cells/mm^2^ Inset g: GCL density (*O. lunatus*) in the periphery of the dorsal retina, less than 2,000 cells/mm^2^ Inset h: GCL density (*O. lunatus*) in the area centralis located centrally, peak of 4,352 cells/mm^2^ Arrowheads in the insets indicate representative cells in the ganglion cell layer. Scale bar equal for all insets. D = dorsal; V = ventral; N = nasal; T =  temporal.

We prepared retinal whole mounts of one eye of each of the individuals in our sample. The other eye was used for tracer injections. As was the case for the visual fields, we found only minor differences between retinas of the same species (up to 10% in total area and total cell count), and large differences between retinas of different species. Considering the few specimens of our sample, here we compare species based upon representative cases only (Figure 2).

We found that *O. degus* had a retinal area of approximately 100 mm^2^, and an estimated total of 297,000 cells in the ganglion cell layer. *O. lunatus* retinal area was similar, about 110 mm^2^, but its estimated total cell number was much lower, about 180,000. Consequently, the overall cell density (cells/retinal area) was lower in *O. lunatus*, specifically 2/3 that of *O. degus* (Table 2). Moreover, the distribution of these cells was not homogeneous. *O. degus* retinal density map shows five isodensity curves, and a well-developed naso-temporally oriented visual streak that runs above the optic nerve head. In contrast, in *O. lunatus* we found only three isodensity curves, and a less developed visual streak, also running above the optic nerve head. In both species cell density declines abruptly towards the dorsal regions of the retina (as shown in Figure 4). Furthermore, we found a circumscribed and well-developed higher cell density region or area centralis (AC.) The size of the AC was relatively similar in both species (*O. degus* 1.0 mm^2^; *O. lunatus* 0.9 mm^2^). AC peak densities were 6,384 cells/mm^2^ in *O. degus* and 4,352 cells/mm^2^ in *O. lunatus*. In *O. degus* the medial AC was located in the temporal part of the retina, 2.8 mm away from the optic nerve head. Interestingly, *O. lunatus* medial AC was located much more centrally, only 0.6 mm away from the optic nerve head (Figure 2, Table 2).

**Table 2:** Summary of the results obtained for *O. degus* and *O. lunatus*.

Species	Lifestyle	Binocularoverlap	Orbitorientation	Cornealdiameter	Peakdensity	Overalldensity	Totalestimation	AC distancefrom ONH	Ipsilateralprojectionto GLd	Ipsilateralprojection to SC
*O. degus*	Diurnal surfacedwelling	50°	41.4°	6.24	6,384	2,990	180,000	2.8 mm	2.75%	0.16%
*O.lunatus*	Nocturnal surfacedwelling	100°	49.8°	7.41	4,352	1,638	108,000	0.6 mm	10.52%	0.95%

Peak density and overall density correspond to cells of the retinal ganglion cell layer. Total estimation of GC was calculated subtracting the theoretical percentage of displaced amacrine cells present in the GCL (we assumed 40%). The ipsilateral projections (last two columns) are shown as percentage relative to the contralateral projection (100%).

AC = area centralis; ONH = optic nerve head; GCL = ganglion cell layer; GC = ganglion cell.

### Central Visual Projections

**Figure 3:**
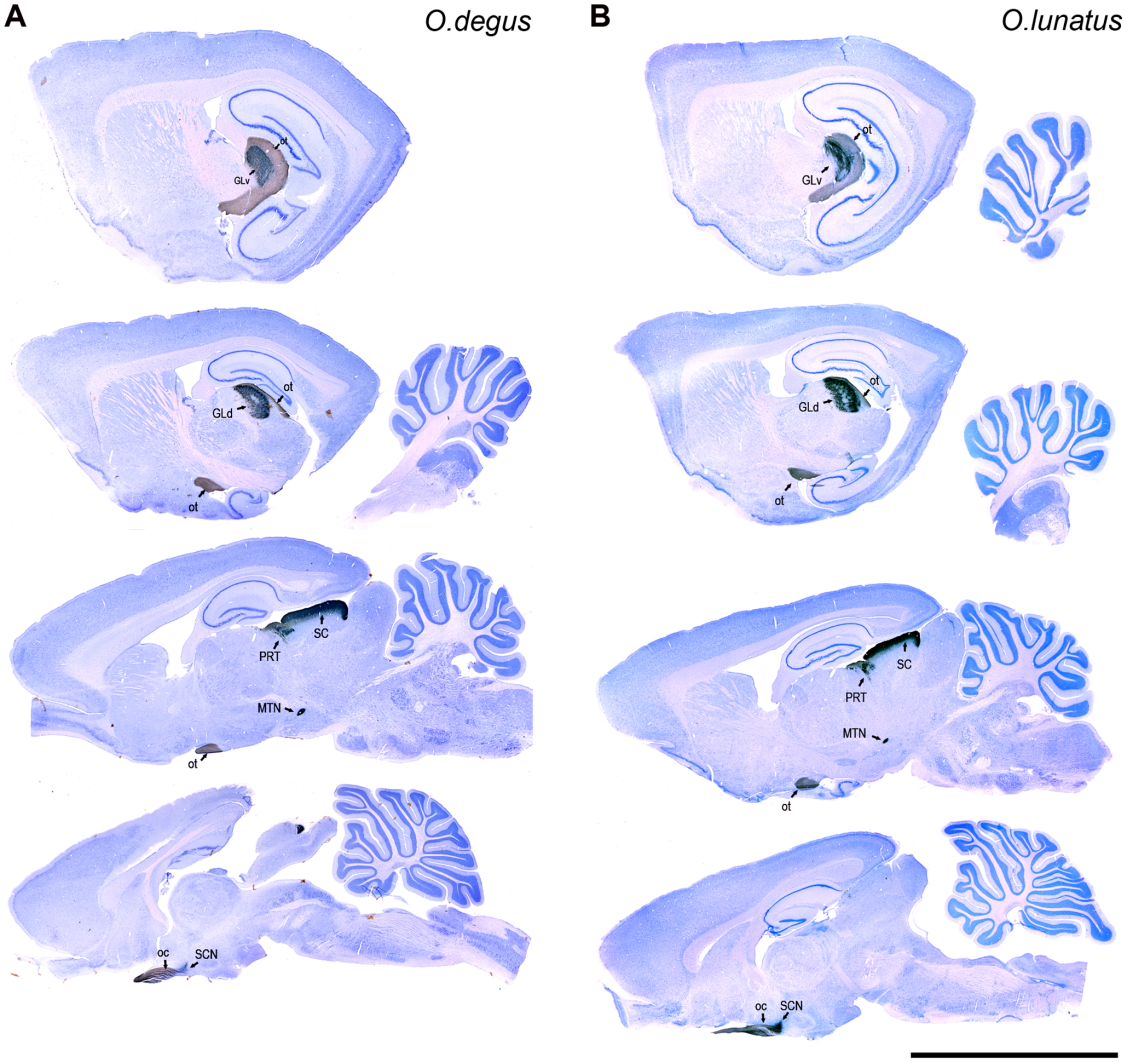
Central visual projections. CTB-labeled retinal fibers and terminals counterstained with Giemsa in sagittal sections (contralateral) in *O. degus* (A) and *O. lunatus* (B). Geniculatus lateralis pars ventralis (GLv); geniculatus lateralis pars dorsalis (GLd); superior colliculus (SC), pretectum (PRT), medial terminal nucleus (MTN); suprachiasmatic nucleus (SCN); optic tract (ot); optic chiasma (oc) Rostral is to the left. Calibration bar = 1 mm.

In both species we found a well-developed visual projection system, with all major mammalian retinal targets easily identifiable: superior colliculus (SC), geniculatus lateralis pars dorsalis (GLd), geniculatus lateralis pars ventralis (GLv), suprachiasmatic nucleus (SCN), pretectal complex (PRT), and accessory optic nuclei (AOS and MTN) (Figure 3). Contralateral and ipsilateral retinal projections were observed in the former four structures, whereas the latter two displayed contralateral projections only.

### Geniculatus Lateralis Pars Dorsalis (GLd)

**Figure 4:**
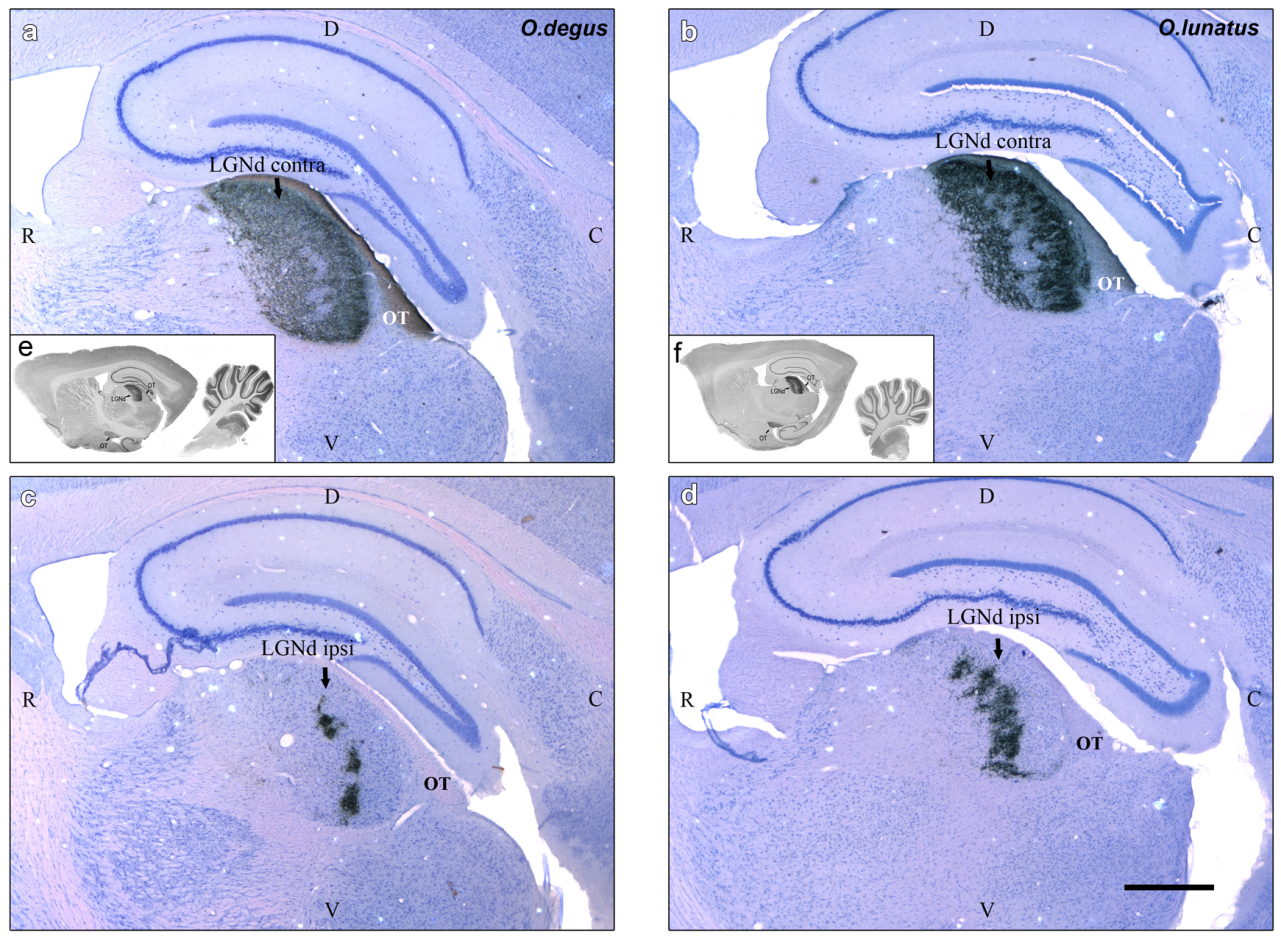
CTB^_^labeled retinal fibers and terminals in GLd. Sagittal sections (contra and ipsilateral) through the geniculatus lateralis pars dorsalis (GLd). a, c (white): *O. degus* (diurnal). b, d (white): *O. lunatus* (nocturnal). Inset: e, f (black) gross section view. Optic tract (OT). Rostral is to the left. Calibration bar = 1 mm.

In the literature, the GLd of rodents is described as a cytoarchitectonically homogeneous structure, with no obvious lamination or subdivisions. However, there are reports of a “hidden” tri-laminar organization in the hooded rat, evidenced mainly by a differential density of retinal terminals [30]–[32]. We found subtle differences between a narrow external (dorsal) lamina, containing coarse retinal terminals, and a broad internal lamina containing finer retinal terminals. On the contralateral side, the external lamina appeared as a continuum of labeled fibers, covering the whole dorsal extension of the nucleus, while the internal lamina showed a clear band lacking labeled retinal fibers (as shown in Figure 4 a,b). This empty zone was located at the centro-caudal third portion of the nucleus. In contrast, on the ipsilateral side most of the GLd appeared free of label, with the exception of a patchy band of labeled terminals with a centro-caudal location within the internal lamina (as shown in Figure 4 c,d). That patchy area corresponded, in shape and position, with the empty contralateral side band.

**Figure 5:**
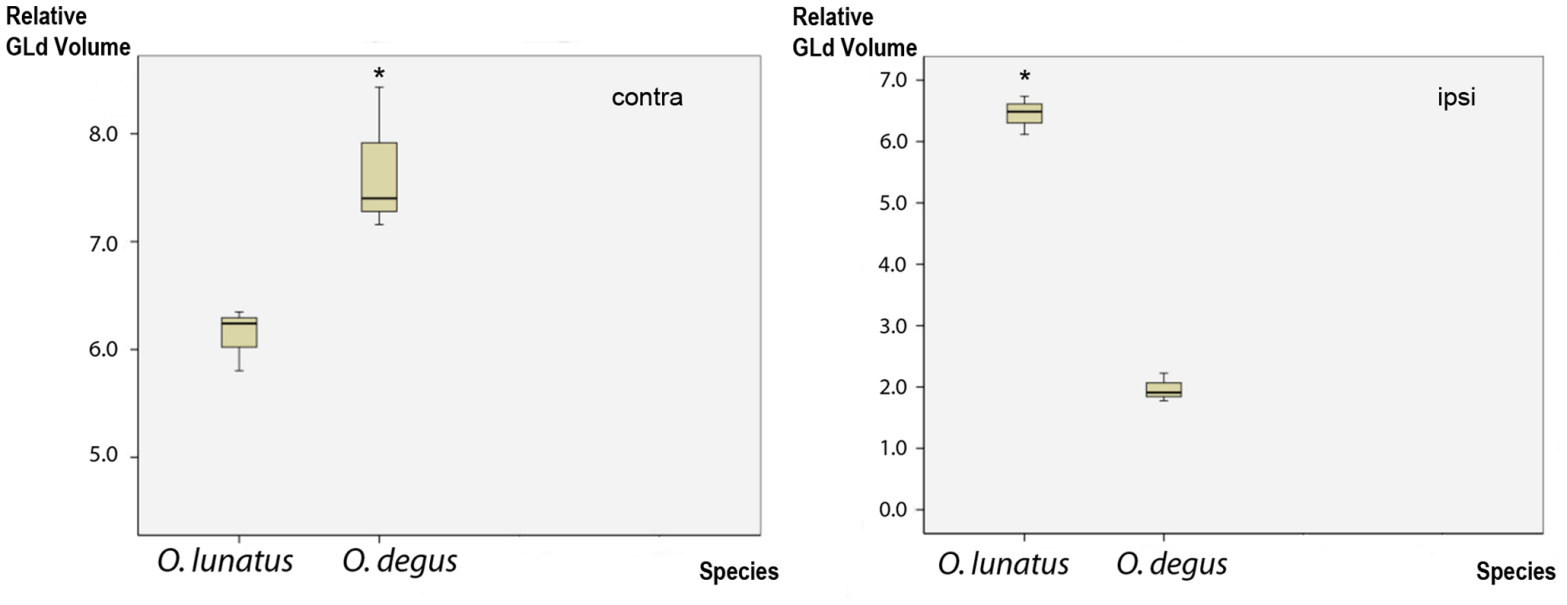
GLd volume. Plots of the contra and ipsilateral GLd volume relative to the brain volume (Y axis) in *O. degus* and *O. lunatus* (X axis). The solid line contained within the box represents the mean. In *GLd contra* relative volume numbers are in multiples of 10^−7^, in *GLd ipsi* 10^−8^. Significant differences are indicated by * *P*≤0.05.

Standardized volumetric analyses of these projections show that the overall volume occupied by the retinal terminals within the contralateral GLd was 20% larger in *O. degus* than in *O. lunatus*, probably reflecting the higher number of ganglion cells present in the diurnal species. In contrast, the volume of the ipsilateral retinal GLd projections of *O. lunatus* was five times larger than that of *O. degus* (Figure 5).

### Superior Colliculus (SC)

**Figure 6:**
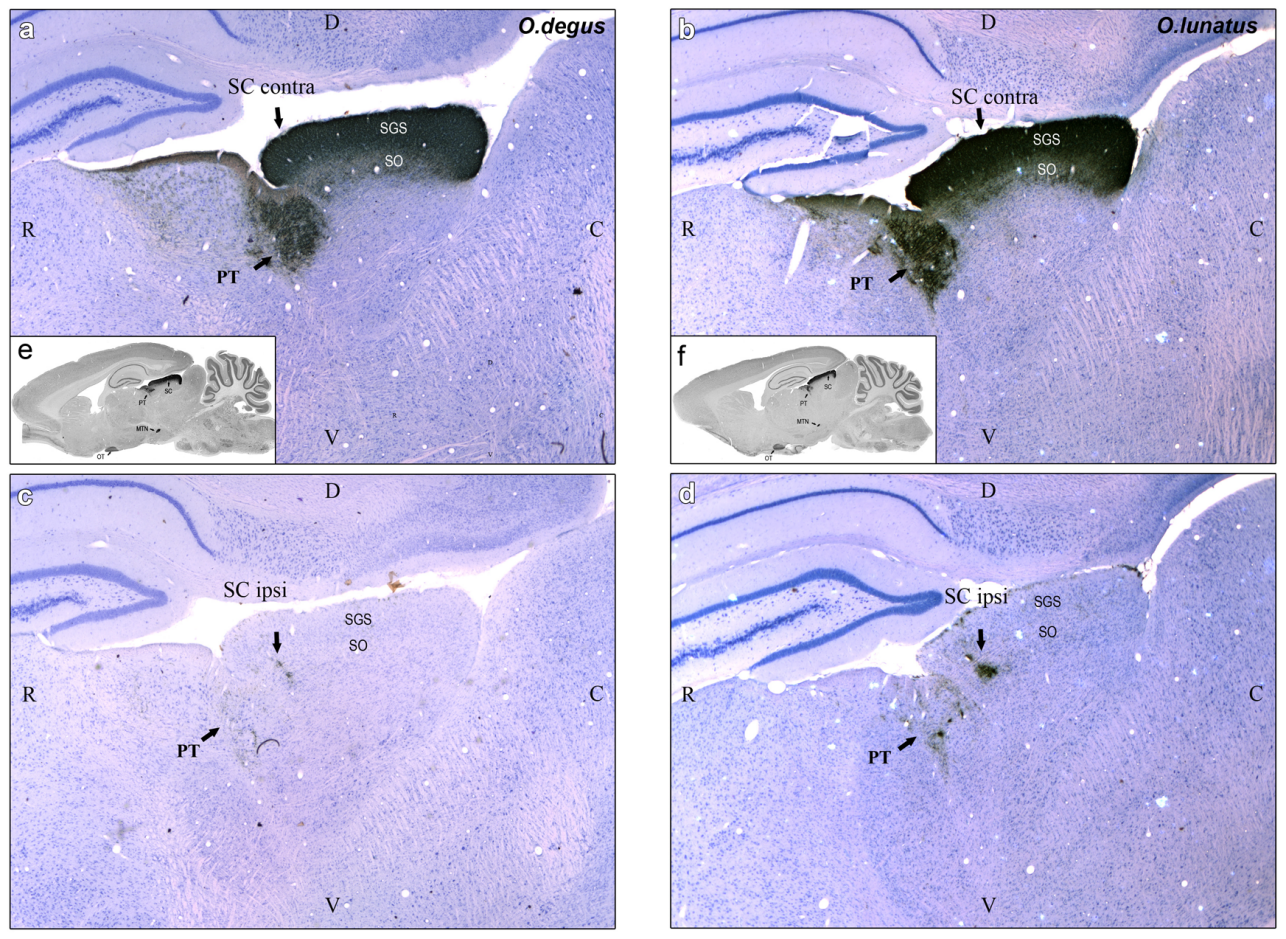
CTB -labeled retinal fibers and terminals in SC. Sagittal sections (contra and ipsilateral) through the superior colliculus (SC). a, c (white): *O. degus* (diurnal). b, d (white): *O. lunatus* (nocturnal). Inset: e, f (black) gross section view. Rostral is to the left. Calibration bar = 1 mm.

In most mammals the SC is the recipient of a massive retinal input featuring two characteristic retinorecipient zones, the external stratum griseum superficiale (SGS), which receives a very dense plexus of retinal arborizations, and the internal stratum opticum (SO), which receives less dense terminal arborizations. Both the SGS and the SO were clearly recognizable in our species (Figure 6). On the contralateral side, both zones appear fully and uniformly covered with retinal terminals (as shown in Figure 6 a,b). In contrast, on the ipsilateral side retinal projections were scarcer, and restricted only to the dorsal portion of the rostral aspect of the SO (as shown in Figure 6 c,d). In all, sagittal, transverse and horizontal sections, the ipsilateral terminals appear as discontinuous patches of coarse labeled processes located close one to each other.

**Figure 7:**
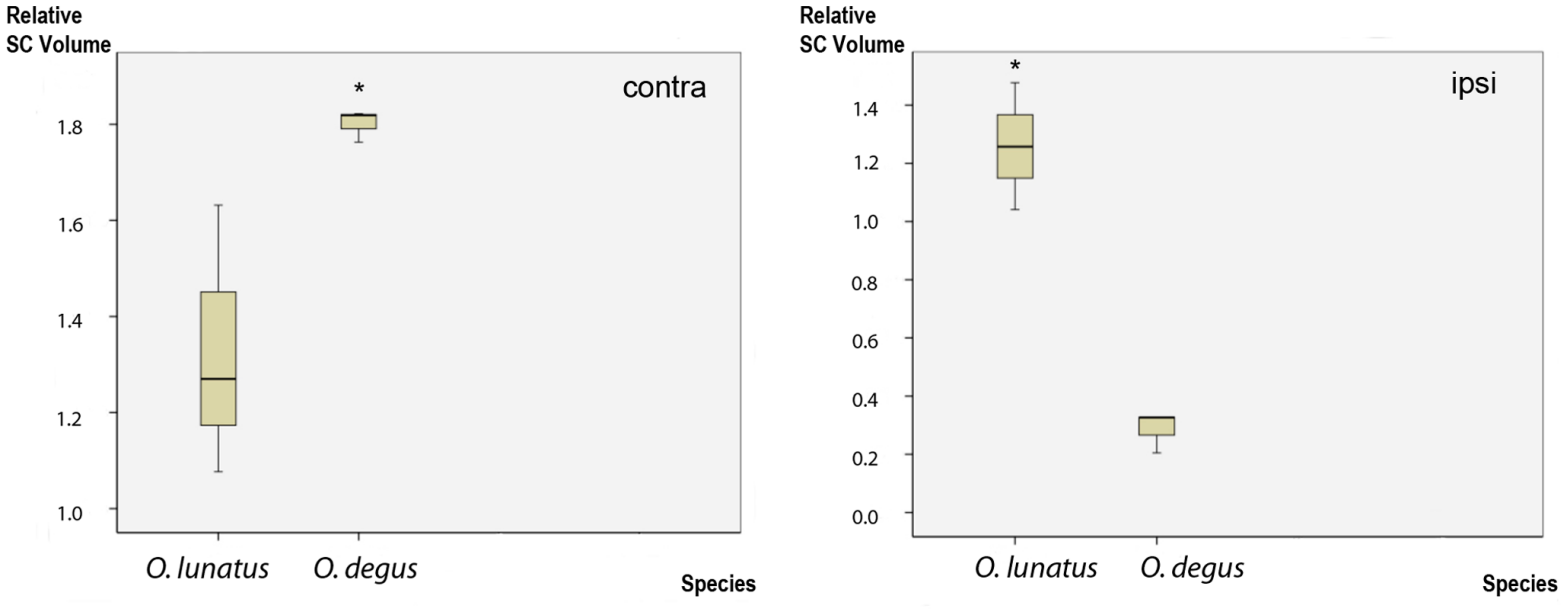
SC relative volume. Plots of the contra and ipsilateral SC volume relative to the brain volume (Y axis) in *O. degus* and *O. lunatus* (X axis). The solid line contained within the box represents the mean. In *SC contra* relative volume numbers are in multiples of 10^−6^, in *SC ipsi* 10^−8^. Significant differences are indicated by * *P*≤0.05.

Quantitative volumetric analyses indicate that the contralateral SC of *O. degus* had a volume 30% larger than that of *O. lunatus*. However, and in close agreement with the above results, the volume of ipsilateral projections was four times larger in *O. lunatus* (Figure 7).

### Projections to other Retinorecipient Nuclei

We also assessed the volume and distribution of retinal terminals in two retinorecipient structures not directly involved with binocular vision: the medial terminal nucleus (MTN) and the suprachiasmatic nucleus (SCN). The MTN provides visual inputs to the neural circuit underlying visuomotor reflexes such as the optokinetic nystagmus [33], while the SCN is the main visual input to the circadian pacemaker [34].

**Figure 8:**
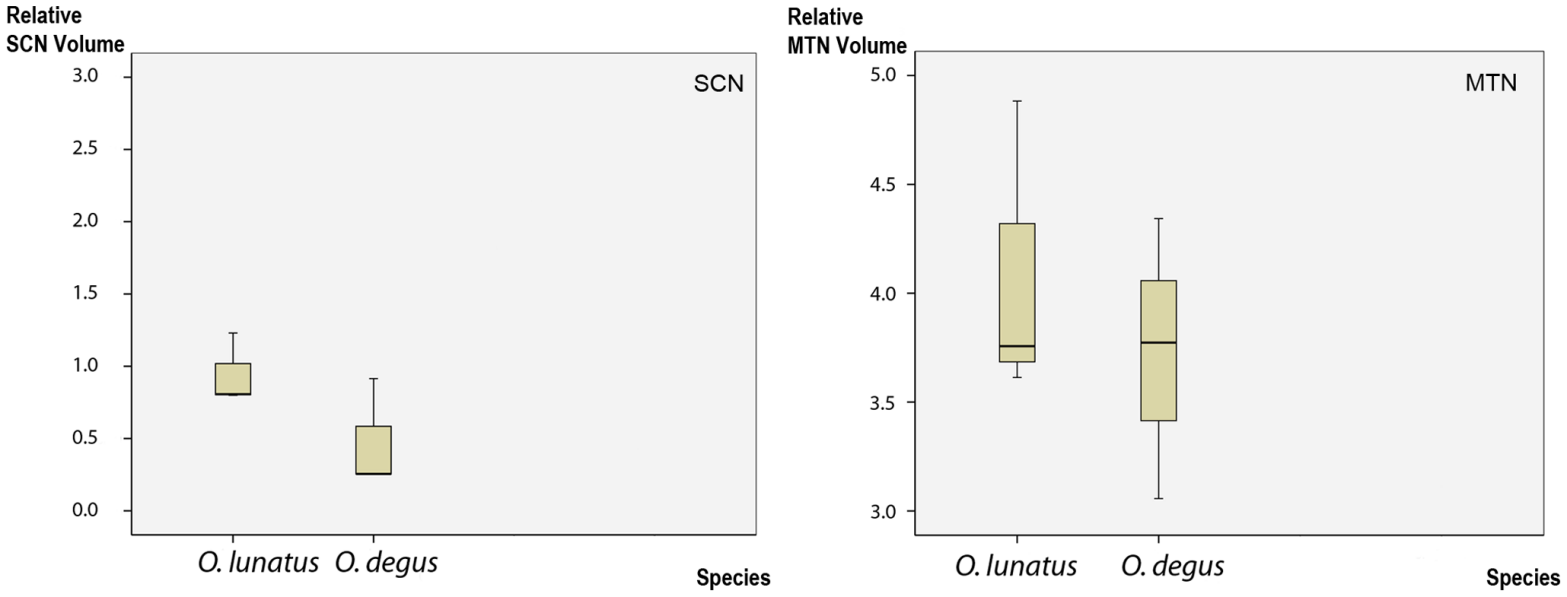
SCN and MTN volume. Plots of the SCN and MTN volume relative to the brain volume (Y axis) in *O. degus* and *O. lunatus* (X axis). The solid line contained within the box represents the mean. In SCN and MTN relative volume numbers are in multiples of 10^−8^.

In both species, retinal-MTN projections were only contralateral, and formed a dense plexus of fine terminals distributed homogeneously through the whole nucleus. In contrast, retinal projections to the SCN were bilateral in both species, and homogeneously covered the nuclei on both sides with a distinctive plexus of less dense, coarse terminals. Volumetric analyses indicated no statistical differences in the volumes of MTN or SCN between *O. degus* and *O. lunatus* (Figure 8).

## Discussion

We found that the visual system of *O. degus* exhibits the characteristic features of a diurnal/crepuscular mammal. Values measured for the visual field size, binocular overlap and orbit convergence were consistent with the values exhibited by other small mammals [1]. The density and distribution of cells in the ganglion cell layer is also comparable to that of other ground dwelling mammals, and fall within the range previously published for another diurnal hystricomorph, the agouti (*Dasyprocta aguti*) [8]. Visual central projections follow the expected pattern for regular small mammals (Muridae, Sciuridae) [31], [35], [36] indicating a well developed but not specialized visual system.

In addition, we found that relative to *O. degus,* the visual system of *O. lunatus* has several features typical of nocturnal mammals, such as a larger corneal diameter and a relative low density of cells in the ganglion cell layer [16], [37] (Figure 2; table 1). *O. lunatus* also has a higher degree of orbit convergence, a corresponding larger monocular and binocular visual field (comparable among rodents to that of the hamster) [1], a more frontally located retinal area centralis, and a larger volume of ipsilateral retinal projections, in particular to the GLd. Even more, our preliminary studies suggest that the primary visual cortex of *O. lunatus* has a larger overall volume, and features a greater number of Layer 4 granular cells, than that of *O. degus* (data not shown). Given that in mammals these latter visual traits constitute the structural basis for functional binocular vision [11], our results point towards the existence of a close evolutionary link between nocturnal visual activity and enhancement of binocularity.

### Binocular Vision: Diurnal vs. Nocturnal

Binocular vision cannot be regarded as an exclusively nocturnal visual trait. In fact, the visual field of most vertebrates, irrespective of their diurnal or nocturnal habits, contains a region of binocular overlap whose extension and position varies according the particular usage of vision in each lifestyle [1], [15]–[17]. Examples of diurnal animals featuring comparatively large binocular overlaps can be found in all vertebrate groups: Binocular overlap varies between 72° to 28° among different species of diurnal predatory batoid fishes (skates and rays), depending upon head morphology and feeding habits [38]. The leopard frog, (*Rana pipiens*), a diurnal insectivorous amphibian, has a binocular overlap of about 90° [39]. Among amniotes, the extremely large binocular field of humans, on one hand, or of the diurnal burrowing owl (*Athene cunicularia)* on the other, can be cited as representative examples. All these cases clearly constitute counterexamples of a purported exclusive association between nocturnal visual habits and binocular enhancement.

Conversely, not all nocturnal species exhibit enhanced binocular overlaps when compared to their diurnal relatives. Striking examples of this can be found in birds: The extent of binocular overlap in the nightjar (*Nyctidromus albicollis*) is comparable to that of regular diurnal birds (approximately 20°) [40], notwithstanding the nocturnal appearance of this animal’s eyes. Other nocturnal predatory birds, such as the night heron (*Nycticorax* spp.), also exhibit “conventional” binocular overlaps of about 22° [41]. Yet, although the association between nocturnal habits and a large binocular overlap is not obligatory, it is of common occurrence in vertebrates, and cannot be regarded as merely coincidental. In addition to the examples mentioned in the introduction, the large binocular overlap and enhanced development of ipsilateral retinal projections found in nocturnal salamanders (Bolitoglossa) can be considered a good example of this tendency [42]. Also, nocturnal insectivorous marsupials such as *Didelphis marsupialia*, *Marmosa mitis* [43]–[45] and *Thylamys elegans* (unpublished results), not only present a large binocular overlap, but a corresponding enlarged development of the ipsilateral retino-thalamic projection.

### The Nocturnal Restriction Hypothesis

In mammals, an extensive ecomorphological study comprising 1300 specimens, performed by Heesy [15], found that the largest degrees of orbit convergence occurred among nocturnal predatory animals, with the highly binocular diurnal primates being the main exception to this rule. However, such studies, as well as other influential ones [4], [11], [46]–[49] do not allow a direct assessment of the relative importance of each of these ecological factors separately. Can each of these factors by themselves lead to an increase in binocularity? In this context, it is worth noting that the results of our study are not influenced by differential phylogenetic constraints, diet type, feeding mode or even locomotor substrate, because both species studied belong to the same genus, have similar diet and foraging habits (herbivorous/folivorous), and are both ground dwelling [50], [51]. Furthermore, both species have raptorial birds (falconiforms, owls) and the fox (*Pseudalopex sp.*) as their main predators [52], [53]. Hence, our study indicates that nocturnal habits *per se*, independently of other factors, result not only in increased orbit convergence, but in an enhancement of all structural features underlying binocular vision.

These results lead us to speculate that the relationship between nocturnal habits and binocular vision may derive solely from the restrictions imposed on vision by the nocturnal environment. This *nocturnal restriction hypothesis,* largely inspired in the *nocturnal predatory hypothesis* previously proposed by Cartmill, Allman and Pettigrew [1], [11], [49] to explain the high degree of binocularity of primates, can be briefly explained as follows: Under nocturnal conditions, the extremely low light levels constitute the main limitation to vision. In most nocturnal animals, this limitation is overcome by incrementing the number of scotopic photoreceptors and decreasing at the same time the number of ganglion cells, so as to maximize the quantum catch efficiency [8], [16], [54], [55]. These changes in cellular composition are also accompanied by an increase in corneal and iris diameter, allowing more light to enter the optic chamber [16], [54]. Corneal enlargement, in turn, determines a homogeneous increase of the visual field size because field size is linearly correlated with the ratio of corneal diameter to axial eye diameter [37], [46]. Due to optical limitations, in a laterally placed eye, an increase of the visual field size will render the temporal binocular crescent of the retina out of focus [15]. A way to attenuate this unwanted outcome would be to increase the degree of convergence of the orbits [15]. Such an increase in orbit convergence, besides extending the binocular overlap, would also lead to a displacement of the retinal area centralis to a more central position, since in mammals the area centralis is always looking at the center of the binocular field [7], [56]. Consequently, the temporal crescent of the retina, which in an hemidecussate animal is the origin of the ipsilateral visual projections, and the magnitude of those projections, would become enlarged.

Thus, according to the nocturnal restriction hypothesis, the enhancement of the structural traits underlying binocular vision observed in many visually active nocturnal mammals would arise as a side effect or a consequence of the nocturnal specialization of the visual system of these animals. The results of our comparison are the expected from such an hypothesis in all aspects: eye size, orbit convergence, visual field size and overlap, retinal specializations and visual central projections. Therefore, the hypothesis seems to have heuristic value, at least in this case, and should be tested in further comparative studies.

It is important to note that the nocturnal restriction hypothesis is meant to apply mainly to mammals, and in particular to the case of diurnal to nocturnal transitions. Nocturnal to diurnal transitions cannot be treated simply as the reverse case of the former. In other words, there is no reason to expect that a nocturnal to diurnal change will result in a decrement of binocular vision. Furthering this notion, Ross [19] shows that the extreme binocularity found in diurnal primates such as humans originates in this lineage’s nocturnal ancestors. He states that subsequently derived diurnal primates retained, and even increased binocularity, because binocular vision is a highly effective visual operation that can be performed equally in diurnal or nocturnal conditions (see below). We think that the same argument can also be applied to other diurnal binocular amniotes, such as the diurnal burrowing owl or the leopard frog.

### Nocturnal Binocular Vision

In mammals, birds and probably other vertebrates, binocular overlap is elaborated centrally to produce binocular fusion, that is, the combination of the visual activity evoked from each of the eyes in a unified and coherent perceptual dimension [11] (see also [40]). A major consequence of binocular fusion is stereopsis, the accurate perception of dimensions of proximate objects, in-depth distance between objects or between borders of an object (solid angle perception), that confers a tridimensional quality to the visual experience. Indeed stereopsis is crucial in many lifestyles, ranging from omnivorous diurnal primates to ground feeding granivorous birds, as it facilitates manipulative visuomotor coordinations as well as object recognition and figure-from-ground (countercamuoflage) segregation (for a review see [57]). An expansion of the binocular overlap such as the one we found in the nocturnal *O. lunatus,* should lead to an extension of the stereoscopic portion of the visual field. However, in our case it is not obvious in which way this expansion may favor nocturnal vision or be favored by it. Stereopsis seems not to be more critical for the visual performance in *O. lunatus* than in *O degus*, since as we stated before, both species share similar visuo-ecological features. In addition, acuity of stereopsis depends upon visual monocular acuity, which in nocturnal species is very limited due to the high degree of convergence of photoreceptors to ganglion cells. Furthermore, the effective range of stereopsis depends upon the spatial separation between the eyes [57], [58]. Hence, such range is inherently limited in octodontine species (probably to less than one meter), and should become even more reduced under dim light conditions. Thus in our case, neither the accuracy nor the range of stereoscopic vision appears to be favored in the nocturnal visual condition.

However, in addition to stereopsis, there are other consequences of binocular fusion that contribute importantly to improve visual performance, especially in nocturnal conditions. Indeed, the popular saying “two eyes see more than one” have solid psychophysical ground. First, binocular summation improves signal-to noise ratio for light detection, by physiologically integrating the activity evoked in corresponding visual loci of each eye [59], [60]. Together with this, binocular summation results in an improvement of light sensitivity and, even more importantly, of contrast sensitivity, both critical parameters for visual operation under dim light conditions [54], [57], [61]. Thus, it seems reasonable to conclude that an expansion of the binocular field such as the one we found in *O. lunatus* indeed entails an extension of the portion of the visual field best suited to nocturnal vision. Further ecological studies will be required to establish whether, how, and to which extent this expanded binocular vision is relevant for this species viability.

### Final Remarks

The results of our comparative study indicate that in mammals the adoption of nocturnal habits *per se*, independently of other relevant visuoecological factors, may be associated with an enhancement of binocular vision, and provides evidence favoring the nocturnal restriction hypothesis. Thus, we regard our results as an invitation to perform more thorough comparative studies, in mammals and other amniotes. In particular, the study of nocturnal predatory birds that lack binocular enhancement, such as the nighthawk, deserves special attention. These birds represent a challenge not only to the nocturnal restriction hypothesis, but also to the alternative notions that link binocularity with predatory behaviors. Furthermore, the recently described case of the New Caledonian crows (*Corvus moneduloides*) [62], diurnal birds from a diurnal lineage that exhibit the largest known degree of binocular overlap among birds (60°), constitute an even more defiant challenge to these notions, and seems to indicate that independently of nocturnality and predatory habits, other factors, in this case manipulative behaviors, may lead to an enhancement of binocularity.
